# Reversal of CSF HIV-1 Escape during Treatment of HIV-Associated Cryptococcal Meningitis in Botswana

**DOI:** 10.3390/biomedicines10061399

**Published:** 2022-06-13

**Authors:** Nametso Kelentse, Sikhulile Moyo, Kesaobaka Molebatsi, Olorato Morerinyane, Shatho Bitsang, Ontlametse T. Bareng, Kwana Lechiile, Tshepo B. Leeme, David S. Lawrence, Ishmael Kasvosve, Rosemary Musonda, Mosepele Mosepele, Thomas S. Harrison, Joseph N. Jarvis, Simani Gaseitsiwe

**Affiliations:** 1 Botswana Harvard AIDS Institute Partnership, Gaborone, Botswana; thobanenametso@gmail.com (N.K.); smoyo@bhp.org.bw (S.M.); kesamolebatsi@gmail.com (K.M.); oloratomorerinyane@gmail.com (O.M.); otbareng@bhp.org.bw (O.T.B.); klechiile@bhp.org.bw (K.L.); tleeme@bhp.org.bw (T.B.L.); david.s.lawrence@lshtm.ac.uk (D.S.L.); rmusonda@bhp.org.bw (R.M.); mosepele.mosepele@gmail.com (M.M.); joseph.jarvis@lshtm.ac.uk (J.N.J.); 2 Department of Medical Laboratory Sciences, Faculty of Health Sciences, University of Botswana, Gaborone, Botswana; kasvosvei@ub.ac.bw; 3 Department of Immunology and Infectious Diseases, Harvard T.H. Chan School of Public Health, Boston, MA 02115, USA; 4 Department of Statistics, Faculty of Social Sciences, University of Botswana, Gaborone, Botswana; 5 Botswana-University of Maryland School of Medicine Health Initiative, Gaborone, Botswana; shatho67@gmail.com; 6 Department of Clinical Research, Faculty of Infectious and Tropical Diseases, The London School of Hygiene and Tropical Medicine, London WC1E 7HT, UK; 7 Department of Internal Medicine, Faculty of Medicine, University of Botswana, Gaborone, Botswana; 8 Centre for Global Health, Institute for Infection and Immunity, St. George’s University of London, London SW17 0RE, UK; tharriso@sgul.ac.uk

**Keywords:** HIV, cryptococcal meningitis, HIV-1 viral load, cerebrospinal fluid (CSF) viral escape, Botswana

## Abstract

Cerebrospinal fluid (CSF) viral escape has been poorly described among people with HIV-associated cryptococcal meningitis. We determined the prevalence of CSF viral escape and HIV-1 viral load (VL) trajectories in individuals treated for HIV-associated cryptococcal meningitis. A retrospective longitudinal study was performed using paired CSF and plasma collected prior to and during the antifungal treatment of 83 participants recruited at the Botswana site of the phase-3 AMBITION-cm trial (2018–2021). HIV-1 RNA levels were quantified then CSF viral escape (CSF HIV-1 RNA ≥ 0.5 log_10_ higher than plasma) and HIV-1 VL trajectories were assessed. CSF viral escape occurred in 20/62 (32.3%; 95% confidence interval [CI]: 21.9–44.6%), 13/52 (25.0%; 95% CI: 15.2–38.2%) and 1/33 (3.0%; 95% CI: 0.16–15.3%) participants at days 1, 7 and 14 respectively. CSF viral escape was significantly lower on day 14 compared to days 1 and 7, *p* = 0.003 and *p* = 0.02, respectively. HIV-1 VL decreased significantly from day 1 to day 14 post antifungal therapy in the CSF but not in the plasma (β = −0.47; 95% CI: −0.69 to −0.25; *p* < 0.001). CSF viral escape is high among individuals presenting with HIV-associated cryptococcal meningitis; however, antifungal therapy may reverse this, highlighting the importance of rapid initiation of antifungal therapy in these patients.

## 1. Introduction

Despite the widespread access to antiretroviral therapy (ART), HIV-associated cryptococcal meningitis remains a significant contributor to AIDS-related mortality mainly in sub-Saharan Africa [1]. Although Botswana has made tremendous strides towards achieving the UNAIDS 90-90-90 targets [2], the incidence of cryptococcal meningitis remains high [3]. During cryptococcal meningitis in immunocompromised individuals, the causative pathogen, *Cryptococcus* spp., and the resultant immune response disrupt the integrity of the blood-brain barrier (BBB) resulting in an influx of pathogens into the central nervous system (CNS) [4,5]. There are still conflicting data on whether HIV coupled with cryptococcal meningitis invades the CNS at a high rate than in HIV-monoinfected individuals [6,7]. 

Some studies have revealed that the interaction between the HIV gp120 and the HIV co-receptors in the human brain microvascular endothelial cells (HBMECs) disrupts the tight junctions, increasing the permeability of the BBB [8,9,10]. The weakened tight junctions then allow HIV to permeate the BBB via infected monocytes, a mechanism known as the “Trojan horse” [11,12]. Consequentially, HIV-1 RNA may be detected in the cerebrospinal fluid (CSF) despite lower or undetectable HIV-1 RNA quantities in the plasma, an event termed CSF viral escape [13]. CSF viral escape has typically been observed in individuals who are on an ART regimen with limited penetration of the CNS through the BBB [14,15,16]. Other factors contributing to CSF viral escape include duration on ART, ART interruptions leading to low-level viremia, and HIV drug-resistance mutations [17,18]. Evidence of CSF viral escape differs among studies with different settings. The prevalence of CSF viral escape may differ between studies depending on the overall ART status of the participants. For instance, one study done on ART-naïve individuals reported a prevalence of 13% (126/972) [19] while another study reported 29% (12/42) cases of CSF viral escape in ART-experienced participants [20]. Furthermore, CSF viral escape may differ in acute versus chronic HIV infection. A study performed on participants with acute HIV-1 infection reported zero cases of CSF viral escape prior to ART initiation [21] contrasting sharply with a 28% prevalence reported in a study with virologically suppressed participants presenting with neurological symptoms [22]. 

Some opportunistic infections such as herpes simplex virus type 2 have been shown to increase HIV-1 VL, with significant decreases following effective treatment [23,24]. However, very little is known about the impact of treating HIV-associated cryptococcal meningitis on HIV-1 VL. Assessing the impact of cryptococcal meningitis treatment on HIV-1 VL may help understand the importance of early initiation of effective antifungal therapy in individuals with HIV-associated cryptococcal meningitis. This study aims to determine the prevalence of CSF HIV-1 viral escape and evaluate the HIV-1 VL trajectories in individuals treated for HIV-associated cryptococcal meningitis. Compared to our previous study which was done prior to the introduction of the “treat all strategy” [25], the current study explores CSF viral escape in a cohort where almost half of the participants were ART-experienced. This study will also advance our understanding of CSF viral escape in the context of antifungal treatment and their impact on CSF and plasma HIV-1 VL over time. To our knowledge, this study is the first to investigate whether a single high dose of liposomal amphotericin B (LAmB) compares to the recommended first-line regimen for treatment of CM in terms of its impact on HIV-1 VL trends. 

## 2. Materials and Methods

### 2.1. Study Design and Population

A retrospective longitudinal study was designed to evaluate HIV-1 VL dynamics in the CSF and plasma of individuals with HIV-associated cryptococcal meningitis. The study included CSF and plasma samples from 83 participants enrolled at the Botswana site of the phase-3 AmBisome Therapy Induction OptimisatioN (AMBITION-cm) randomised controlled trial which was conducted between 2018 and 2021. AMBITION-cm compared a single high-dose of LAmB given alongside 14 days of flucytosine and fluconazole with the World Health Organisation recommended first-line regimen at the time which was one week of amphotericin B deoxycholate with flucytosine followed by fluconazole. The primary outcome was all-cause mortality at ten weeks [26]. We included CSF and plasma with at least one sample collected from either day 1 (prior to antifungal therapy) or days 7 and 14 of antifungal treatment. These CSF and plasma samples were either paired or unpaired depending on availability.

### 2.2. Ethics Statement

This study was reviewed and approved by the University of Botswana ethics committee and a research permit was granted by the Health Research and Development Council (HRDC) at the Botswana Ministry of Health (approval number: HPDME 13/18/1). All participants from the AMBITION-cm clinical trial had previously consented to the use of their stored samples for future research projects and the study was approved by the HRDC (ISRCTN number: ISRCTN72509687).

### 2.3. Laboratory Methods

All CSF samples were centrifuged at 1200× *g* for 10 min to obtain cell-free supernatant. Available CSF and plasma HIV-1 RNA levels from days 1, 7 and 14 were quantified using COBAS^®^ AmpliPrep/COBAS^®^ TaqMan^®^ HIV-1 Test, v2.0 (Hoffmann-La Roche, Basel, Switzerland) with a limit of detection of 20 copies/mL. Samples with insufficient volumes were diluted using phosphate-buffered saline (PBS) resulting in a 3-fold dilution. Clinical data and demographics collected in the primary study were used. 

### 2.4. Outcome Definitions and Covariates

CSF viral escape was defined as CSF HIV-1 RNA ≥ 0.5 log_10_ higher than plasma [13]. We then determined factors associated with CSF viral escape using the following covariates: pleocytosis, CD4+ T-cell count, CSF protein and glucose concentration, duration on ART and the available demographics. Pleocytosis was defined as CSF white cell count (WCC) ≥ 20 cells/mm^3^ [27,28,29]. The cut-off to identify elevated protein concentration was ≥1 g/L [22]. Glucose concentration was dichotomised as <2 mmol/L and ≥2 mmol/L, with <2 mmol/L indicating hypoglycorrhachia [30]. We categorised ART status into ART-naïve, ART-experienced for <6 months and ≥6 months.

For the determination of HIV-1 VL trends over time, the outcome of interest was CSF and plasma HIV-1 VL. The covariates in this analysis included gender, CSF fungal load, age, treatment arm, CD4+ T-cell count, mental status, and CSF WCC. We adjusted for the different ART decisions made at participant enrolment. The ART decisions were categorised into three groups: ART-naïve, ART-interrupted, and ART-continued. The ART decisions were based on the guidelines for the management of ART-exposed patients recruited to the AMBITION-cm study [31]. Briefly, for ART naïve participants, ART was started 4 to 6 weeks after enrolment. ART was stopped if it had been very recently initiated and could have resulted in an IRIS or if had been a regimen prescribed for greater than 6 months and therefore deemed either to be ineffective or not being taken. The exception to this was where there was documentation of viral suppression at the time of admission, in which instance ART was continued. Finally, ART was continued if the participant has been reported to be adherent to ART for ≤ 6 months. 

### 2.5. Statistical Analysis

Baseline demographics, clinical, and laboratory variables were summarized using descriptive statistics (counts with percentages and medians with interquartile ranges [IQR]). The HIV-1 VL and fungal load were log-transformed before performing statistical tests. We used binomial proportions to estimate prevalence. Wilcoxon signed-rank test was used to compare median HIV-1 VL for paired samples. Factors associated with baseline CSF viral escape were determined using Firth logistic regression which accounts for a small sample size. To minimise potential selection bias due to missing data we used Multivariate Imputation by Chained Equations (MICE) to impute incomplete multivariate data under the assumption of missing at random (MAR). Five imputed datasets were created. This model considered the following observed covariates; gender, age, ART status at baseline, ART decision, CSF fungal load, CSF WCC, CD4+ T-cell count, treatment arms, duration on ART, and CSF protein and glucose concentration. The participants who had missing data due to death also had missing data being imputed by the model. We then employed the generalized estimating equations (GEE) model to determine factors associated with HIV-1 VL changes over the three study time points. This model accounts for the correlation of repeated measurements within a participant while examining potential time trends. All statistical analyses were performed using R version 4.1.2 and a *p*-value of < 0.05 was considered statistically significant.

## 3. Results

### 3.1. Baseline Participant’s Characteristics

Table 1 summarises the baseline clinical characteristics and demographics of the participants in our study. Of the 83 participants, 57 (68.7%) were male, the median age was 40 (IQR 34–44) years, and 37/83 (44.6%) were ART-experienced with a median ART duration of 18 (IQR 0–67) months. Sixteen participants (43.2%) on ART were on Tenofovir Disoproxil Fumarate (TDF), Lamivudine (3TC) and Dolutegravir (DTG) as a single tablet regimen. The median CD4+ T-cell count was 30 (IQR 9–62) cells/µL and the median CSF WCC was 7.5 (IQR 2–59) cells/mm^3^. At baseline, the median CSF and plasma HIV-1 VL were 4.3 (IQR 3.4–5.5) and 5.0 (IQR 3.9–5.4) log_10_ copies/mL, respectively.

### 3.2. Comparison of Paired CSF and Plasma HIV-1 VL at Days 1, 7 and 14

**Figure 1:**
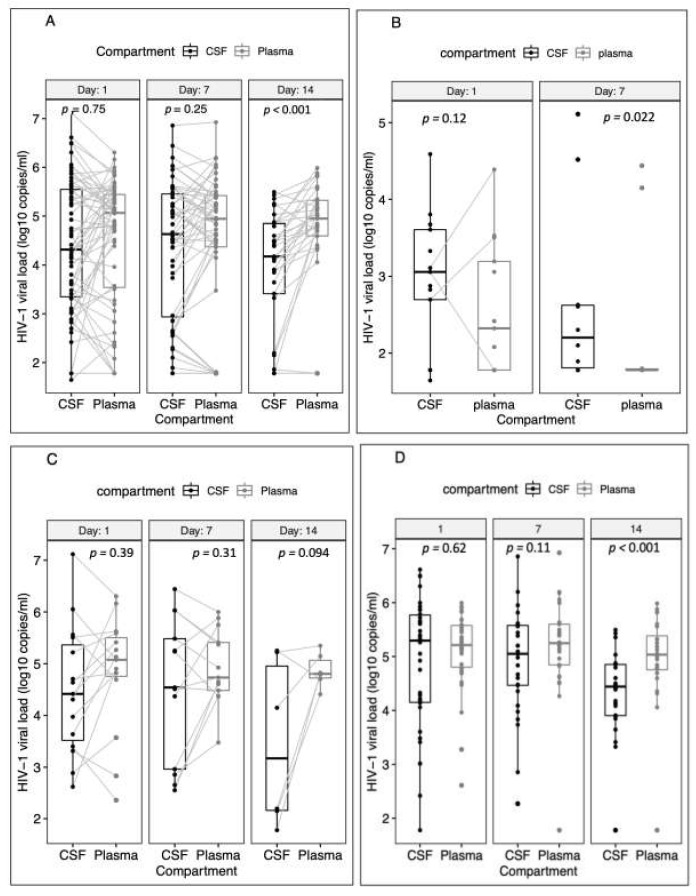
Comparison of median HIV-1 viral load in matched CSF and plasma samples on days 1, 7 and 14 of the study. (**A**) Overall results with all ART groups combined. (**B**) ART-continued group. (**C**) ART-interrupted group. (**D**) ART-naïve group. We could not compute the analysis for day 14 in the ART-continued group because of the small sample size.

Of the 83 participants, 62 had paired CSF/plasma day 1 samples, 52 had day 7 and 33 had day 14 samples. Samples were missing either due to the participant dying before sample collection or due to low sample volume or particularly if the CSF opening pressure was too low, and a few declining lumbar punctures (Figure S1). Although plasma was higher than CSF HIV-1 VL at all timepoints, a statistically significant difference was observed on day 14 only with plasma and CSF HIV-1 VL medians of 4.9 (4.6–5.3) and 4.2 (3.4–4.9) log_10_ copies/mL respectively (*p* < 0.001), Figure 1A. We also compared the paired CSF and plasma HIV-1 VL on different days in the ART-continued, ART-interrupted, and ART-naïve categories (Figure 1B–D). CSF HIV-1 VL was significantly higher than plasma HIV-1 VL on day 7 but not on day 1 in the ART-continued group, *p* = 0.02 (Figure 1B). We could not analyse data for day 14 due to the small sample size. There was no significant difference between the CSF and plasma HIV-1 VL at all time points in the ART-interrupted category (Figure 1C). In the ART-naïve group, we observed a significantly higher plasma than CSF HIV-1 VL on day 14 (*p* < 0.001), Figure 1D.

### 3.3. Prevalence and Factors Associated with CSF HIV-1 Viral Escape

CSF viral escape was found in 20/62 (32.3%; 95% confidence interval [CI]: 21.9% to 44.6%), 13/52 (25.0%; 95% CI: 15.2% to 38.2%) and 1/33 (3.0%; 95% CI: 0.16% to 15.3%) for baseline, day 7 and 14 samples, respectively. The proportion of CSF viral escape cases for day 14 was lower than on day 1 and day 7, *p* = 0.003 and *p* = 0.02, respectively. At baseline, there were 5/78 (6.4%) and 4/66 (6.1%) participants with plasma and CSF HIV-1 VL of <60 copies/mL, respectively. Of those with both samples, 2/62 had HIV-1 VL of < 60 copies/mL in both the CSF and plasma. In participants experiencing CSF viral escape, 3/20 (15.0%) had HIV-1 VL of < 60 copies/mL in the plasma and > 60 in the CSF (Table S1). Due to the 3-fold dilution factor used which yielded a limit of detection of 60 copies/mL, we could not conclude that these individuals were suppressed or had undetectable HIV-1 VL. 

In a model adjusted for CD4+ T-cell count, CSF fungal load, duration on ART and glucose concentration, pleocytosis remained an independent predictor of CSF viral escape with 6.5-fold higher odds (95% CI, 1.1–36.9, *p* = 0.034) in those with WCC ≥ 20 cells/µL, Table 2. The odds of having CSF viral escape were 4.4-fold (95% CI, 1.3–14.7, *p* = 0.015) higher in individuals with protein concentration ≥1 g/L; however, we could not include both protein concentration and CSF WCC a priori because of multicollinearity between these variables. The odds of having CSF viral escape were 80% lower in individuals with CD4+ T-cell count < 50 cells/µL; however, the response was the opposite, and the association was lost in a multivariable analysis (*p* = 0.683) possibly due to multicollinearity. ART status, duration on ART, CSF viral escape, and CD4+ T-cell count were not associated with mortality or mental status (data not shown).

### 3.4. HIV-1 Viral Load Trajectories and Factors Associated with Changes in HIV-1 Viral Load

**Figure 2:**
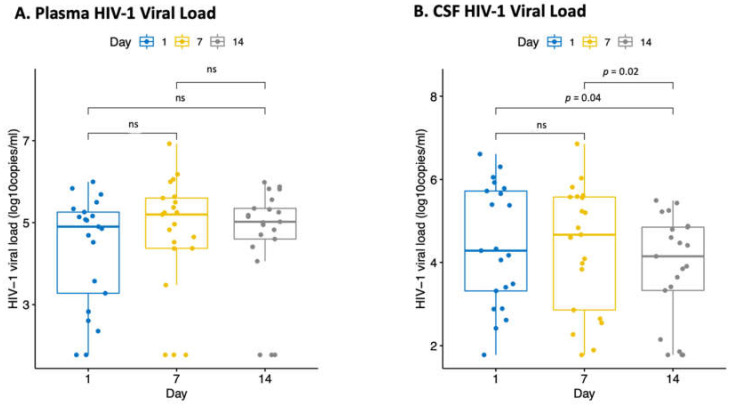
(**A**) Boxplots comparing median plasma HIV-1 viral load between days 1, 7 and 14 for each participant with complete follow-up data (combining all the three treatment decision groups). (**B**) Boxplots comparing median CSF HIV-1 viral load between days 1, 7 and 14 for each participant with complete follow-up data (combining all the three treatment decision groups).

We performed a descriptive analysis of HIV-1 VL for the combined treatment decision groups at different time points by compartment. We observed no statistically significant difference between days 1, 7 and 14 plasma HIV-1 VL (Figure 2A). In the CSF, day 14 HIV-1 VL was significantly lower than day 1 and 7 HIV-1 VL, *p* = 0.04 and *p* = 0.02, respectively (Figure 2B). We further analysed the CSF and plasma HIV-1 viral load trends for everyone with complete follow-up data categorised by the three treatment decisions (Figure S2). In most individuals, the CSF HIV-1 VL decreased over time compared to the plasma HIV-1 VL for the ART interrupted and ART-naïve groups while the ART continued group did not have enough data to draw any conclusion. The results from the multivariable model (Table 3) show that an increase in time by two weeks (from day 1 to day 14) was associated with a significant decrease in the CSF HIV-1 VL (β= −0.47, 95% CI: −0.69 to −0.25, *p* < 0.001). Although a decrease was also observed from day 1 to day 7, this was not statistically significant. An insignificant increase in the plasma HIV-1 VL from day 1 to day 7 and day 1 to day 14 was observed, *p* = 0.133 and *p* = 0.746, respectively. Pleocytosis was a predictor of an increase in CSF but not plasma HIV-1 VL, holding all other variables constant (β = 0.62; 95% CI, 0.18 to 1.06; *p* = 0.006).

Since the ART decisions were made based on the prediction of viremia, we compared median HIV-1 VL to determine if they correspond with the ART decisions. The median plasma HIV-1 VL in the ART-continued group was 2.3 (1.8–3.3) log_10_ copies/mL while the ART-interrupted and the ART-naïve groups were 5.0 (4.6–5.4) and 5.2 (4.8–5.6) log_10_ copies/mL, respectively. The plasma HIV-1 VL in the ART-continued group was significantly lower than the ART-interrupted and ART-naïve group, *p* < 0.001. We then assessed whether there was an association between ART decisions and changes in HIV-1 VL over time (Table 3). When compared to the ART-continued group, the ART-interrupted and ART-naïve group were associated with a significant increase in CSF HIV-1 VL, (β = 1.38; 95% CI, 0.68 to 2.08; *p* < 0.001) and (β = 1.69; 95% CI, 0.98 to 2.40; *p* < 0.001), respectively. The association for plasma HIV-1 VL also followed the same direction in the ART-interrupted (β = 2.64; 95% CI, 2.14 to 3.14, *p* < 0.001) and ART-naïve group (β = 2.83; 95% CI, 2.37 to 3.30, *p* < 0.001). In the univariable model, being on ART for less than 6 months was associated with a decrease in both CSF (β = −1.35; 95% CI, −1.87 to −0.82; *p* < 0.001) and plasma (β = −2.25; 95% CI, −2.74 to −1.76; *p* < 0.001) HIV-1 VL while in the ART-naïve group, the decrease was not statistically significant. However, this could not be included in the multivariable analysis due to multicollinearity with the ART decisions. In this multivariable analysis, there was no significant difference in the control and the single-dose treatment arms in reducing CSF and plasma HIV-1 over time, (β = 0.005; 95% CI, −0.47 to 0.46; *p* = 0.983) and (β = 0.04; 95% CI, −0.47 to 0.56; *p* = 0.873), respectively. 

We also assessed CSF and plasma HIV-1 VL changes over time in the ART-naïve group alone (Table 4). We observed a significant decrease in CSF HIV-1 VL from day 1 to day 14 (β = −0.66; 95% CI, −0.92 to 0.41; *p* < 0.001). In the plasma, HIV-1 VL increased significantly from day 1 to day 7 (β = 0.22; 95% CI, 0.02 to 0.42; *p* < 0.031) while from day 1 to day 14 the increase was not statistically insignificant.

## 4. Discussion

HIV viral escape in the CNS has been well documented in individuals with HIV-associated neurocognitive disorders (HAND); however, there are conflicting reports on whether this is also seen in individuals with HIV-associated opportunistic infections such as cryptococcal meningitis [7,32,33,34]. In this study that compared CSF HIV-1 VL versus plasma HIV-1 VL in individuals with HIV-associated cryptococcal meningitis, we observed a substantial proportion of individuals experiencing CSF viral escape (32.3% with day 1 samples, 25.0% with day 7 and 3.0% with day 14 samples). This is higher than our previous study which observed a very low prevalence (2.9%) of CSF viral escape among 34 individuals with HIV-associated cryptococcal meningitis [25]. This may be because the previous study analysed a combination of samples collected on either day 3, 7, or 14 post-initiation of antifungal therapy, potentially missing episodes of CSF viral escape which only occurred around baseline. In keeping with previous research findings, we found that CSF pleocytosis and elevated CSF protein concentration were associated with CSF viral escape [14,35,36] and this may be explained by CNS inflammation and immune activation due to an altered BBB leading to leakage of proteins and CNS infiltration by HIV-infected cells [37]. We did not observe a significant difference between the two treatment arms in relation to changes in the HIV-1 VL indicating that the single high-dose LAmB regimen is as effective as the standard regimen in reducing the HIV VL in the CSF.

The HIV-1 VL was comparable between the compartments at the first two time points but higher in plasma than CSF on day 14, mainly due to a decrease in CSF HIV-1 VL. One study reported significantly higher HIV-1 VL in plasma than CSF at all three-time points [38]. In another study where the CSF and plasma HIV-1 VL were compared on days 1 and 14, plasma HIV-1 VL was reported to be higher than CSF HIV-1 VL on both days [39]. Our results may be supported by reports that show that *Cryptococcus* enhances HIV replication in the CNS causing CSF HIV-1 RNA levels to approach that of the plasma or even exceed them [40,41]. On day 14 however, the CSF HIV-1 VL may be lowered by the effectiveness of the cryptococcal meningitis treatment [40]. 

To better understand the impact of cryptococcal meningitis antifungal therapy on HIV-1 VL, we assessed the HIV-1 VL trajectories over the three-time points. We observed a significant reduction in CSF HIV-1 VL from day 1 to day 14 which was not seen in plasma. Since antifungals can eliminate the *Cryptococcus spp.* which is known to enhance HIV replication and the CNS being the main site of infection for cryptococcal meningitis, the reduction of HIV VL may be more significant locally than systemically. The integrity of the BBB may be restored once antifungal therapy is introduced, lowering the trafficking of HIV-infected cells into the CNS, and ultimately decreasing the CSF HIV-1 VL. Pleocytosis was a predictor of an increase in CSF HIV-1 VL over time, indicating a continuous influx of HIV-infected cells in the CNS [15]. The absence of ART during this period was also associated with an increase in CSF and plasma HIV-1 VL showing that antifungals alone do not reduce CSF HIV-1 VL as much as when coupled with ART. In a model where we assessed the trends in HIV-1 VL over time in ART-naïve individuals only, we observed a significant decrease in the CSF HIV-1 VL and not plasma from day 1 to day 14 suggesting that the treatment of cryptococcal meningitis has positive effects on the CSF HIV-1 VL even in the absence of ART.

Several limitations to this study need to be acknowledged. In terms of time under observation, 11/83 participants died before day 14; therefore, our conclusion about the reversal of escape or decrease in HIV-1 VL due to antifungal therapy should be further evaluated in larger studies. Although we lost these individuals, it is highly unlikely that the reversal of escape and a decrease in HIV-1 VL were associated with death on day 14. We used MICE under the assumption of MAR to account for missing data; however, selection bias could have been introduced since we could not rule out the possibility of data missing not at random (MNAR), especially in cases of death. We used a very conservative definition of CSF viral escape, and many investigators would now consider any HIV-1 VL higher in the CSF than plasma CSF viral escape [42,43]. Another limitation of our study was the lack of a control group without cryptococcal meningitis infection to help understand the CSF HIV viral escape in the absence of cryptococcal meningitis. However, CSF viral escape has also been reported in individuals without neurological symptoms at the acute HIV infection stage. CSF viral escape did not develop even after months of ART suggesting that ART initiation at the early course of infection may help prevent the development of CSF viral escape [21]. Symptomatic CSF viral escape has also been reported in individuals on ART; one study showed that a switch to ARVs with a better CNS penetration effectiveness suppresses the HIV-1 VL and resolves the neurologic symptoms [44]. Lastly, we could not evaluate the impact of ART on compartmentalisation because almost half of the individuals on ART were recently initiated.

## 5. Conclusions

Taken together, our results suggest that the integrity of the BBB may be disrupted in individuals with HIV-associated cryptococcal meningitis causing elevated HIV-1 RNA in CNS and a high prevalence of CSF viral escape. The HIV-1 VL could be reversible with effective cryptococcal meningitis antifungal therapy even in the absence of ART, indicating the possibility of CSF viral escape not persisting post-cryptococcal meningitis infection. These results highlight the importance of rapid initiation of effective antifungal therapy in individuals with HIV-associated cryptococcal meningitis.

## Figures and Tables

**Table 1:** Baseline characteristics of the 83 participants studied.

Characteristic	Value (*n* = 83)
Age, years, median (IQR)	40 (34–44)
Gender, Male, No. (%)	57 (68.7%)
CD4+ T-cell count, cells/µL, median (IQR) *	30 (9–62)
ART status, No. (%)	
ART-naïve	46 (55.4)
ART-experienced	37 (44.6)
Duration on ART, months, median (IQR)	18 (0–67)
ART-regimen, No. (%)	
ABC/3TC + DTG	1 (2.7)
AZT/3TC + EFV	2 (5.4)
AZT/3TC + NVP	1 (2.7)
DTG; Other	1 (2.7)
TDF/3TC/DTG	16 (43.2)
TDF/3TC/EFV	13 (35.1)
Unknown	3 (8.1)
ART-decision, No. (%)	
ART-continued	16 (19.3)
ART-interrupted	21 (25.3)
ART-naïve	46 (55.4)
CSF WCC, cells/mm^3^, median (IQR) *	7.5 (2–59)
CSF protein concentration, g/L, median (IQR) *	0.69 (0.52–1.40)
CSF glucose concentration, mmol/L, median (IQR) *	2.4 (1.5–3.0)
CSF Fungal load, log_10_ CFU/mL, median (IQR)	4.8 (3.4–5.7)
Baseline HIV-1 viral load, log_10_ copies/mL, median (IQR) *	
CSF	4.3 (3.4–5.5)
Plasma	5.0 (3.9–5.4)

Abbreviations: 3TC, Lamivudine; ABC, Abacavir; ART, antiretroviral therapy; AZT, Zidovudine, CFU, colony-forming units; CSF, cerebrospinal fluid; DTG, Dolutegravir; EFV, Efavirenz; IQR, interquartile range; NVP, Nevirapine, TDF, Tenofovir Disoproxil Fumarate; WCC, white cell count. * 21 patients were missing baseline CD4+ T-cell count, 3 patients were missing CSF WCC, 18 were missing CSF protein concentration, 3 were missing CSF glucose concentration, 17 were missing baseline CSF HIV-1 VL and 3 were missing baseline plasma HIV-1 VL.

**Table 2:** Factors associated with CSF HIV-1 viral escape prior to antifungal therapy.

Characteristic	CSF Viral Escape		Univariable Analysis		Multivariable Analysis *	
Characteristic	Yes (*n* = 20) No. (%), Median (IQR)	No (*n* = 42) No. (%), Median (IQR)	OR (95% CI)	*p*-Value	Adjusted OR (95% CI)	*p*-Value
Fungal burden, log_10_ CFU/mL	3.9 (1.7–5.3)	5.3 (4.2–5.8)	0.9 (0.8–1.0)	0.056	0.9 (0.8–1.2)	0.640
Age, years						
≥35	12 (60)	30 (71.4)	0.6 (0.2–1.8)	0.365	-	-
<35	8 (40)	12 (28.6)	1.0 (Ref)	-	-	-
Gender						
Male	13(65)	29 (69.0)	0.8 (0.3–2.5)	0.731	-	-
Female	7 (35)	13 (31.0)	1.0 (Ref.)	-	-	-
CD4, cells/µL						
<50	4 (30.8)	24 (75.0)	0.2 (0.04–0.6)	**0.007**	1.6 (0.2–13.8)	0.683
≥50	9 (69.2)	8 (25.0)	1.0 (Ref.)	-	1.0 (Ref.)	-
Missing	7 (35.0)	10 (23.8)				
Duration on ART						
On ART ≥6 months	4 (23.5)	11 (26.2)	1.1 (0.3–3.9)	0.942	2.5 (0.4–15.2)	0.328
On ART <6 months	7 (41.2)	6 (14.3)	3.1 (0.9–11.2)	0.083	3.0 (0.3–27.8)	0.354
ART-naïve	9 (52.9)	25 (59.5)	1.0 (Ref.)	-	1.0 (Ref.)	-
CSF protein concentration, g/L						
≥1	10 (55.6)	7 (21.2)	4.4 (1.3–14.7)	**0.015**	-	-
<1	8 (44.4)	26 (78.8)	1.0 (Ref.)	-	-	-
Missing	2 (10)	9 (21.4)				
CSF glucose concentration, mmol/L						
<2	11 (55.0)	14 (35.0)	2.2 (0.8–6.5)	0.144	1.9 (0.4–8.6)	0.401
≥2	9 (45.0)	26 (65.0)	1.0 (Ref.)	-	1.0 (Ref.)	-
Missing	-	2 (4.8)				
CSF WCC, cells/mm^3^						
≥20	12 (60.0)	10 (25.0)	4.3 (1.4–13.1)	**0.009**	6.5 (1.1–36.9)	**0.034**
<20	8 (40.0)	30 (75.0)	1.0 (Ref.)	-	1.0 (Ref.)	-
Missing	-	2 (4.8)				

ART, antiretroviral therapy; CSF, cerebrospinal fluid; WCC, white cell count; OR, odds ratio. * We adjusted for all variables with a *p*-value of <0.2 under a univariable model except for protein concentration due to collinearity with CSF WCC.

**Table 3:** Factors associated with changes in the CSF and plasma HIV-1 viral load over time (*n* = 83).

	CSF				PLASMA			
	Univariable		Multivariable		Univariable		Multivariable	
	β Coefficient (95% CI)	*p*-Value	β Coefficient (95% CI)	*p*-Value	β Coefficient (95% CI)	*p*-Value	β Coefficient (95% CI)	*p*-Value
Day:								
Day 7	−0.03 (−0.21, 0.15)	0.718	−0.03 (−0.21, 0.15)	0.718	0.12 (0.04, 0.28)	0.133	0.12 (−0.04, 0.28)	0.133
Day 14	−0.47 (−0.69, −0.25)	**<** **0.001**	−0.47 (−0.688, −0.252)	**<** **0.001**	0.03 (−0.15, 0.21)	0.746	0.03 (−0.15, 0.21)	0.746
ART Decision:								
ART-interrupted	1.32 (0.70, 1.95)	**<** **0.001**	1.38 (0.68, 2.08)	**<** **0.001**	2.48 (2.05, 2.92)	**<** **0.001**	2.64 (2.14, 3.14)	**<** **0.001**
ART-naïve	1.67 (1.16, 2.17)	**<** **0.001**	1.69 (0.98, 2.40)	**<** **0.001**	2.62 (2.22, 3.02)	**<** **0.001**	2.83 (2.37, 3.30)	**<** **0.001**
Age (≥35)	0.31 (−0.22, 0.85)	0.252	-	-	0.29 (−0.31, 0.90)	0.344	-	-
Gender (Male)	0.29 (−0.22, 0.81)	0.265	-	-	−0.04 (−0.61, 0.53)	0.895	-	-
CSF WCC (≥20)	0.37 (−0.11, 0.85)	0.134	0.62 (0.18, 1.06)	**0.006**	−0.33 (−0.90, 0.25)	0.265	-	-
Baseline Fungal load, log_10_ CFU/mL	0.10 (−0.04, 0.23)	0.157	−0.03 (−0.17, 0.10)	0.618	0.24 (0.08, 0.40)	**0.004**	0.01 (−0.06, 0.08)	0.773
Treatment arm (Single dose)	0.005 (−0.47, 0.46)	0.983	-	-	0.04 (−0.47, 0.56)	0.873	-	-
CD4 (<50), cells/µL	0.64 (0.11, 1.17)	**0.017**	0.28 (−0.32, 0.88)	0.356	1.02 (0.39, 1.65)	**0.002**	−0.28 (−0.63, 0.06)	0.108
CSF protein concentration (≥1), g/L	0.29 (−0.24, 0.81)	0.286	-	-	−0.30 (−0.92, 0.32)	0.346	-	-
Duration on ART:								
On ART (<6 months)	−1.35 (−1.87, −0.82)	**<** **0.001**	- *	-	−2.25 (−2.74, −1.76)	**<** **0.001**	-	-
ART-naïve	−5.5 (−1.12, 0.03)	0.061	- *	-	−0.33 (−0.82, 0.17)	0.194	-	-
Abnormal mental status	0.23 (−0.28, 0.73)	0.384	0.38 (−0.004, 0.76)	0.052	0.04 (−0.52, 0.59)	0.893	0.24 (−0.03, 0.52)	0.086

ART, antiretroviral therapy; CSF, cerebrospinal fluid; WCC, white cell count; OR, odds ratio. * We adjusted for all variables with a *p*-value of <0.2 under a univariable model except for protein concentration due to collinearity with CSF WCC. A dash (-) means data not computed because of an insignificant *p*-value in a univariable model.

**Table 4:** Factors associated with changes in CSF and plasma HIV-1 over time in ART-naïve participants (*n* = 46).

	CSF				PLASMA			
	Univariable		Multivariable		Univariable		Multivariable	
	β Coefficient (95% CI)	*p*-Value	β Coefficient (95% CI)	*p*-Value	β Coefficient (95% CI)	*p*-Value	β Coefficient (95% CI)	*p*-Value
Day:								
Day7	7.21 × 10^−5^ (−0.27, 0.27)	1.000	7.21 × 10^−5^ (−0.27, 0.27)	1.000	0.22 (0.02, 0.42)	**0.031**	0.22 (0.02, 0.42)	**0.031**
Day14	−0.66 (−0.92, −0.41)	**<0.001**	−0.66 (−0.92, −0.41)	**<** **0.001**	0.02 (−0.17, 0.21)	0.822	0.02 (−0.17, 0.21)	0.822
Age (≥35)	−0.005 (−0.52, 0.51)	0.983	-	**-**	0.24 (−0.23, 0.70)	0.326	-	-
Gender (Male)	−0.23 (−0.64, 0.19)	0.291	-	-	−0.14 (−0.50, 0.22)	0.448	-	-
CSF WCC (≥20)	0.62 (0.25, 1.00)	**0.0** **01**	0.52 (−0.03, 1.07)	0.065	0.21 (−0.14, 0.57)	0.242	**-**	-
Baseline Fungal load, log_10_ CFU/mL	−0.04 (−0.26, 0.18)	0.716	-	-	−0.10 (−0.17, −0.02)	**0.016**	−0.04 (−0.13, 0.05)	0.417
Treatment arm (Single dose)	0.11 (−0.36, 0.57)	0.658	-	-	0.40 (0.06, 0.74)	**0.021**	0.35 (0.05, 0.65)	**0.022**
CD4 (<50), cells/µL	−0.08 (−0.76, 0.60)	0.814	-	-	−0.52 (−0.87, −0.16)	**0.005**	−0.25 (−0.60, 0.09)	0.153
CSF protein concentration (≥1), g/L	0.56 (0.07, 1.05)	**0.025**	0.16 (−0.52, 0.83)	0.649	0.46 (0.15, 0.78)	**0.004**	0.19 (−0.13, 0.52)	0.247
Abnormal mental status	0.43(0.02, 0.85)	**0.042**	0.38 (−0.08, 0.83)	0.104	0.23 (−0.08, 0.54)	0.141	0.11 (−0.16, 0.38)	0.414

Abbreviations: ART, antiretroviral therapy; CSF, cerebrospinal fluid; WCC, white cell count; OR, Odds ratio. In a Multivariable Model, adjustments were made for all variables with a *p*-value under 0.2.

## Data Availability

Data is contained within the article and supplementary material.

## Supplementary Materials

The following supporting information can be downloaded at: https://www.mdpi.com/article/10.3390/biomedicines10061399/s1, Figure S1: Study flow chart showing the number of participants with available and missing samples. Samples were missing either due to the participant dying before collection before the end of day 14 or due to low sample volume or low CSF opening pressure and a few declining lumbar punctures. * Samples were not included because the CSF was collected on any day other than day 7 (2 samples) or day 14 (3 samples). A total of 24 participants had CSF samples available at all time points while 60 participants had complete plasma samples. The remaining participants had at least one sample missing; Figure S2: A. CSF HIV-1 viral load trends for each participant with complete follow-up data categorised by the three treatment decisions. B. Plasma HIV-1 viral load trends for each participant with complete follow-up data categorised by the three treatment decisions. Table S1: Characteristics of Individuals with CSF viral escape at baseline.
