# Supplementary material for: Reversal of CSF HIV-1 Escape during Treatment of HIV-Associated Cryptococcal Meningitis in Botswana
Source: Biomedicines. 2022 Jun 13;10(6):1399. doi: 10.3390/biomedicines10061399 (PMC9219642; doi:10.3390/biomedicines10061399)
Supplement: Supplementary file 1 [file biomedicines-10-01399-s001.zip › Supplementary Files/Figure S1.pdf]

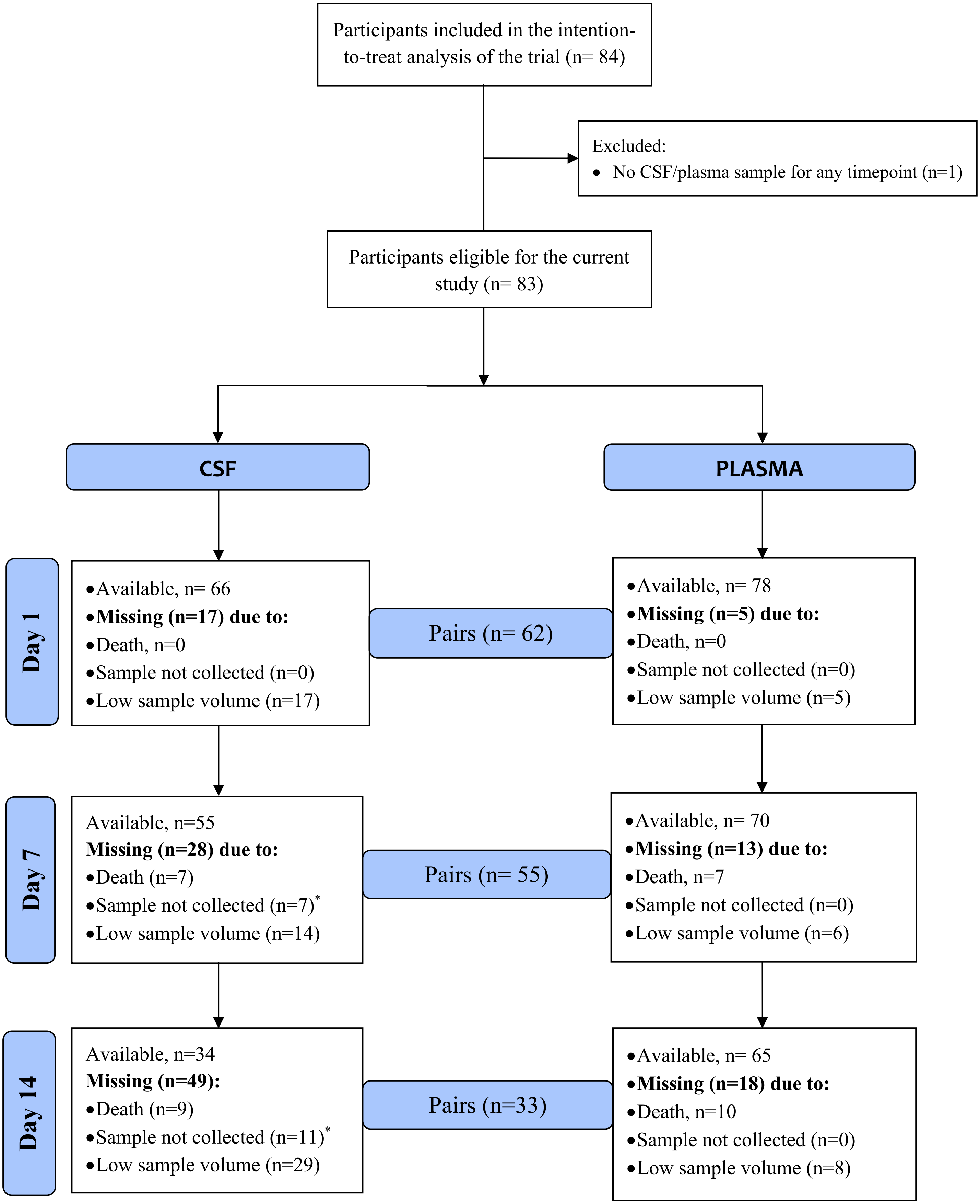

Figure S1: Study flow chart showing the number of participants with available and missing samples. Samples were missing either due to the participant dying before collection the end of day 14 or due to low sample volume or due to low CSF opening pressure and a few declining lumbar punctures. *Samples were not included because the CSF was collected on any day other that day 7 (2 samples) or day 14 (3 samples). A total of 24 participants had CSF samples available at all timepoints while 60 participants had complete plasma samples. The remaining participants had at least one sample missing.
