# Supplementary material for: Reversal of CSF HIV-1 Escape during Treatment of HIV-Associated Cryptococcal Meningitis in Botswana
Source: Biomedicines. 2022 Jun 13;10(6):1399. doi: 10.3390/biomedicines10061399 (PMC9219642; doi:10.3390/biomedicines10061399)
Supplement: Supplementary file 1 [file biomedicines-10-01399-s001.zip › Supplementary Files/Table S1.pdf]

Table S1: Characteristics of Individuals with CSF viral escape at baseline

ID	Sex	Age (Years)	Mental Status	On ART*	Duration on ART (months)	ART Regimen	Baseline CD4 count (cells/ µL)	Fungal load (CFU/mL)	CSF WCC (cells/mm^3)	CSF Glucose (mmol/L)	CSF Protein (g/L)	Mortality Outcome	CSF HIV-1 VL (copies/ml)	Plasma HIV-1 VL (copies/ml)
**1**	M	40	NORMAL	Yes	3	TDF/3TC/DTG	87	165	275	0.80	3.65	Alive	1290	<60
**2**	F	34	NORMAL	No	N/A	N/A	73	8300	160	1.60	1.65	Alive	526450	137724
**3**	M	38	NORMAL	Yes	34	TDF/3TC/EFV	10	50	10	2.70	0.63	Alive	362670	49275
**4**	M	31	ABNORMAL	N/A	N/A	N/A	N/A	2650000	5	1.90	0.97	Alive	736076	127701
**5**	M	39	NORMAL	Yes	112	TDF/3TC/EFV	63	1465000	130	1.60	0.86	Alive	1129270	119019
**6**	M	61	NORMAL	Yes	1	TDF/3TC/DTG	N/A	15	130	3.10	1.19	Alive	4064	<60
**7**	M	44	ABNORMAL	N/A	N/A	N/A	N/A	45	7	3.10	1.10	Alive	3162300	809064
**8**	M	34	ABNORMAL	N/A	N/A	N/A	N/A	8450	8	2.60	N/A	Alive	3087332	186726
**9**	F	56	ABNORMAL	Yes	0	TDF//3TC/DTG	N/A	31000	70	1.70	0.55	Alive	2524	228
**10**	M	31	NORMAL	Yes	0	TDF//3TC/DTG	84	41500	8	2.10	0.87	Alive	670	120
**11**	F	24	NORMAL	N/A	N/A	N/A	8	3750	50	2.40	1.08	Alive	2019112	145347
**12**	F	34	ABNORMAL	Yes	18	TDF//3TC/DTG	339	37500	200	1.60	2.30	Alive	753	<60
**13**	M	38	NORMAL	Yes	2	TDF//3TC/DTG	190	10	85	0.90	1.63	Alive	38864	3393
**14**	M	35	ABNORMAL	Yes	109	TDF/3TC/EFV	51	15	145	1.00	1.18	Alive	13200792	2019123
**15**	F	33	NORMAL	N/A	N/A	N/A	33	550000	130	1.20	1.49	Alive	4102696	684588
**16**	M	44	NORMAL	N/A	N/A	N/A	N/A	750000	5	3.10	0.57	Alive	990370	283332
**17**	M	41	ABNORMAL	N/A	N/A	N/A	43	530000	4	1.30	0.76	Dead	238140	71499
**18**	F	50	NORMAL	Yes	0	TDF/3TC/DTG	295	20	62	3.00	0.52	Dead	4730	1143
**19**	F	41	ABNORMAL	No	N/A	N/A	N/A	153000	10	3.30	N/A	Dead	846132	113559
**20**	M	31	ABNORMAL	Yes	0	TDF/3TC/EFV	127	70	80	1.40	2.10	Dead	6356	1566

Abbreviations: 3TC, Lamivudine; ART, antiretroviral therapy; CFU, colony-forming units; CSF, cerebrospinal fluid; DTG, Dolutegravir; EFV, Efavirenz; F, female; IQR, interquartile range; M, male; N/A, not available or not applicable; TDF, Tenofovir Disoproxil Fumarate; WCC, white cell count. *ART status is N/A because the individuals were newly diagnosed with HIV.
