# Supplementary figures and images for: Reversal of CSF HIV-1 Escape during Treatment of HIV-Associated Cryptococcal Meningitis in Botswana
Source: Biomedicines. 2022 Jun 13;10(6):1399. doi: 10.3390/biomedicines10061399 (PMC9219642; doi:10.3390/biomedicines10061399)

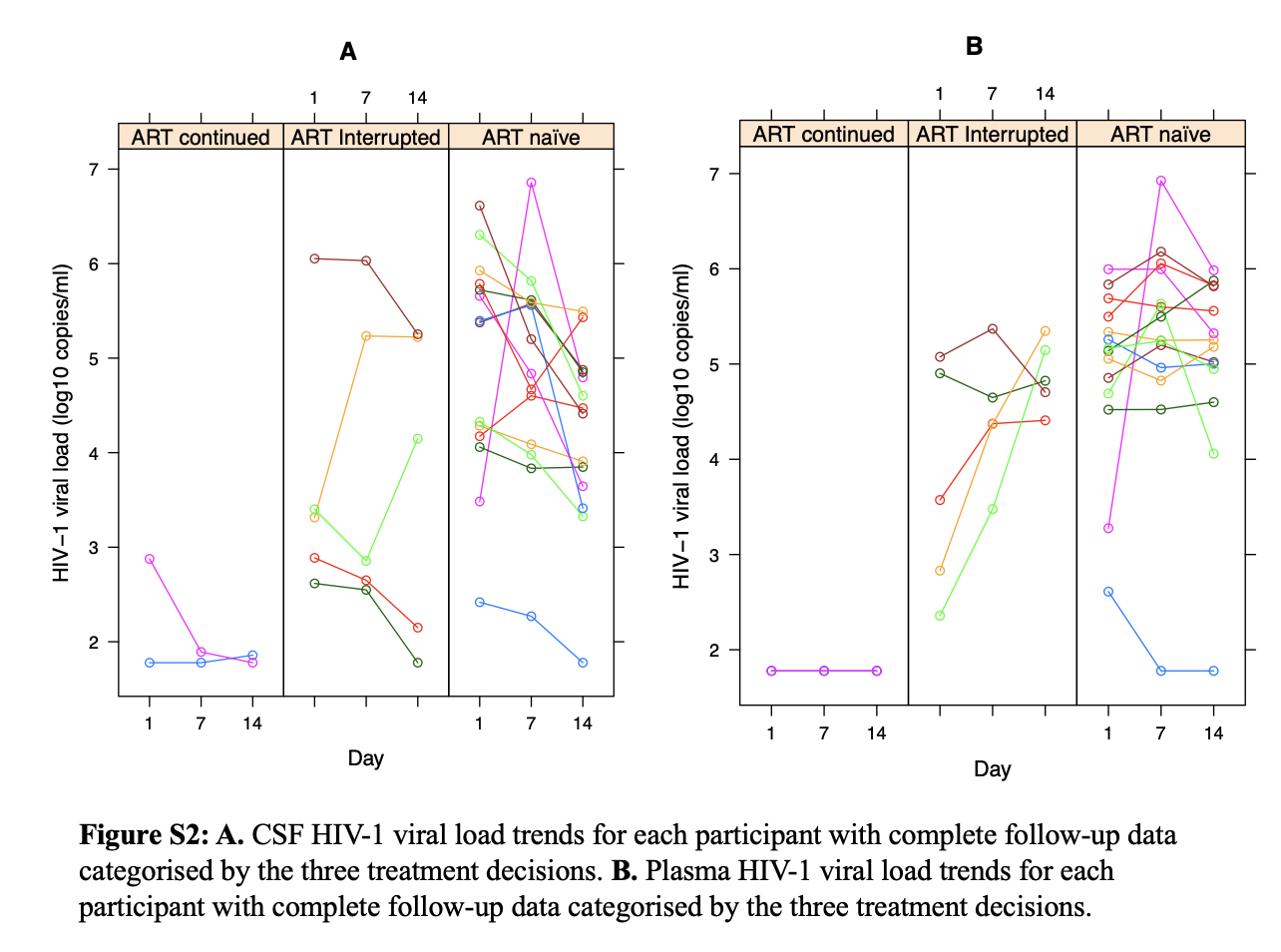

Supplement: Supplementary file 1 [file biomedicines-10-01399-s001.zip › Supplementary Files/Figure S2.png]
